# Theory of topological insulator waveguides: polarization control and the enhancement of the magneto-electric effect

**DOI:** 10.1038/srep43115

**Published:** 2017-02-21

**Authors:** J. A. Crosse

**Affiliations:** 1 Department of Electrical and Computer Engineering, National University of Singapore, 4 Engineering Drive 3 117583, Singapore

## Abstract

Topological insulators subject to a time-reversal-symmetry-breaking perturbation are predicted to display a magneto-electric effect that causes the electric and magnetic induction fields to mix at the material’s surface. This effect induces polarization rotations of between ≈1–10 mrad per interface in an incident plane-polarized electromagnetic wave normal to a multilayered structure. Here we show, theoretically and numerically, that by using a waveguide geometry with a topological insulator guide layer and magneto-dielectric cladding it is possible to achieve rotations of ≈100 mrad and generate an elliptical polarization with only a three-layered structure. This geometry is beneficial, not only as a way to enhance the magneto-electric effect, rendering it easier to observe, but also as a method for controlling the polarization of electromagnetic radiation.

Topological insulators^1^,^2^ are time-reversal-symmetric materials that display non-trivial topological order, which results in an insulating bulk and protected conducting edge states^3^. Such behaviour has been observed in Group V/VI alloys that have strong enough spin-orbit coupling to induce band inversion, e.g. Bi_1−x_Sb_x_^4^,^5^, Bi_2_Se_3_, Bi_2_Te_3_ and Sb_2_Te_3_^6^,^7^,^8^. Owing to it’s unusual electronic properties, topological insulators have also been predicted to display a magneto-electric effect which causes the electric, **E**, and magnetic induction, **B**, fields to mix at the material’s surface^9^,^10^. This effect will induce Kerr (reflection) and Faraday (transmission) rotations in plane polarized light, with previous studies predicting a ≈1–10 mrad rotation in an incident electromagnetic wave normal to a single topological-insulator-magneto-dielectric interface^11^,^12^,^13^,^14^,^15^.

The magneto-electric effect is induced by applying a time-reversal-symmetry-breaking perturbation to a topological insulator’s surface. This can be done either by introducing magnetic dopants^10^ or by applying an external magnetic field^12^. Such a perturbation opens a gap in the surface states the of size of which is proportional to the perpendicular magnetic field or the 2D surface dopant density^16^ - current experiments in Bi_2_Se_3_ have observed a ≈50 meV gap with 16% Fe dopants^17^ and a ≈100 meV gap with 22% Cr dopants^18^, with even larger gaps predicted. Within this gap, the resulting quantum Hall effect^9^,^10^ leads to modified constitutive relations for the material


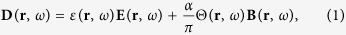



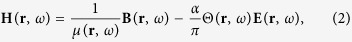


where *ε*(**r**, *ω*) and *μ*(**r**, *ω*) are the usual permittivity and permeability and Θ(**r**, *ω*) is the axion coupling which takes odd values of *π* in a time-reversal-symmetry-broken topological insulator and even values of *π* in a conventional magneto-dielectric with, *α*, the fine structure constant. For convenience we work in natural units with *c* = *ε*_0_ = *μ*_0_ = 1. The lowest Hall plateau, which is generated by the strongest magnetic perturbation, leads to Θ(**r**, *ω*) = *π*. Reducing the magnet perturbation will not increase the axion coupling until the next Hall plateau is reached. Thus, axion couplings of Θ(**r**, *ω*) = *π*, 3*π*, 5*π*, … are only accessible with magnetic perturbation on the order of Tesla. Higher plateaus are accessible at lower fields but these plateaus are narrower and can be difficult to resolve. Thus, although this effect is achievable with an infinitesimal field, strong fields may be required to observe the discrete nature axion coupling. Maxwell’s equations are unchanged by the axion coupling, however, the modified constitutive relations result in a term proportional to ∇Θ(**r**, *ω*) × **E**(**r**, *ω*) appearing in the wave equation^19^,^20^. Thus, gradients in the axion coupling cause the electric field to rotate in the plane perpendicular to that gradient and, hence, plane polarized electromagnetic radiation will undergo a rotation in regions of spatially varying axion coupling.

Faraday and Kerr rotations in have been observed in THz spectroscopy studies of Bi_2_Te_3_^21^,^22^,^23^,^24^ and strained HgTe^25^,^26^,^27^. As the mechanism by which these rotations occur is directly related to the topological surface states of these materials, the effect can be a used as a probe. Studies have sought to measure the Landau level structure^21^,^22^, surface states^23^ and transport properties^25^,^26^ of these materials using the Faraday and Kerr rotations, as well as the band spin splitting due to an external magnetic field^24^. In term of application, these materials show promise for room temperature control of the phase and polarization of THz radiation^27^.

Another feature of the magneto-electric, unique to topological insulators, is the universal quantization of the Faraday rotation. Previous theoretical studies have predicted that, for plane-polarized light normal to a topological insulator surface in either the thin film geometry, where the wavelength of the incident radiation is much greater than the thickness of the topological insulator slab, *λ* ≫ *d*, or the resonant case in the thick film geometry, where the film thickness is equal to a half integer number of wavelengths, *d* = *Nλ*/2, the Faraday rotation is quantized in integer units of the fine structure constant, *α*^12^,^13^,^14^,^15^. This feature distinguishes topological insulators from materials that exhibit the magneto-optical Kerr effect, which can also be used to induce Faraday rotations in waveguides^28^. This effect has recently been experimentally observed^29^,^30^,^31^. However, this universality is only occurs for normal incident light in the stated geometries and, although the Faraday rotation is still quantized, the magnitude of the rotation deviates from *α* for non-resonant thick film geometries or non-normal incident angles^32^.

There are advantages for trying to increase the Faraday rotation. As a surface state probe, an increase in the Faraday rotation would render small features of the states easier to observe and hence provide higher sensitivity for these precision measurements. In terms of applications, enhancement of the effect could lead to a large enough rotation for signal processing, thus, providing a method for controlling the polarization of THz radiation. It is possible to increase the rotation simply by adding more layers^26^,^32^. However, with each additional layer the fabrication process becomes increasingly difficult. In addition, in order to obtain consistent results, any precision experiment wishing to study a topological insulator would require that all the surfaces in a multi-layered structure to be in the same state (i.e. to have the same gating potential and experience the same fields), which can sometimes be challenging. Here, we show that it is possible to significantly enhance the magneto-electric effect using a waveguide, thus rendering the effect easier to detect whilst only using a three layered structure. In this geometry, the radiation probes only two surfaces, which is advantageous for spectroscopic measurements. Furthermore, a waveguide is a standard photonic component and provides an easily fabricable structure for on-chip THz radiation control.

## Theory

Here, we study a slab waveguide geometry, layered in the y-direction with optical modes propagating in the x-direction, consisting of a homogeneous time-reversal-symmetry-broken topological insulator guide layer and a homogeneous conventional magneto-dielectric cladding. Thus, the axion coupling only varies at the interfaces between the guide layer and the cladding. Such a waveguide supports four types of mode, transverse electric odd and even modes (TE) with the electric field, **E**(**r**, *ω*), polarized in the z-direction and transverse magnetic odd and even modes (TM) with the magnetic field, **H**(**r**, *ω*), polarized in the z-direction. As the effect of the axion coupling is to rotate the polarization of the propagating field, one would expect the TE and TM polarizations to mix. Thus, one should observe a rotation in the polarization of a plane-polarized electromagnetic wave as it propagates down the waveguide. Furthermore, as the propagation constants of the TE and TM modes differ in a waveguide, one should also observe a changing ellipticity. From a geometric optics perspective, one can view the effect as a series of successive Kerr rotations from the multiple reflections at the waveguide interfaces.

**Figure 1:**
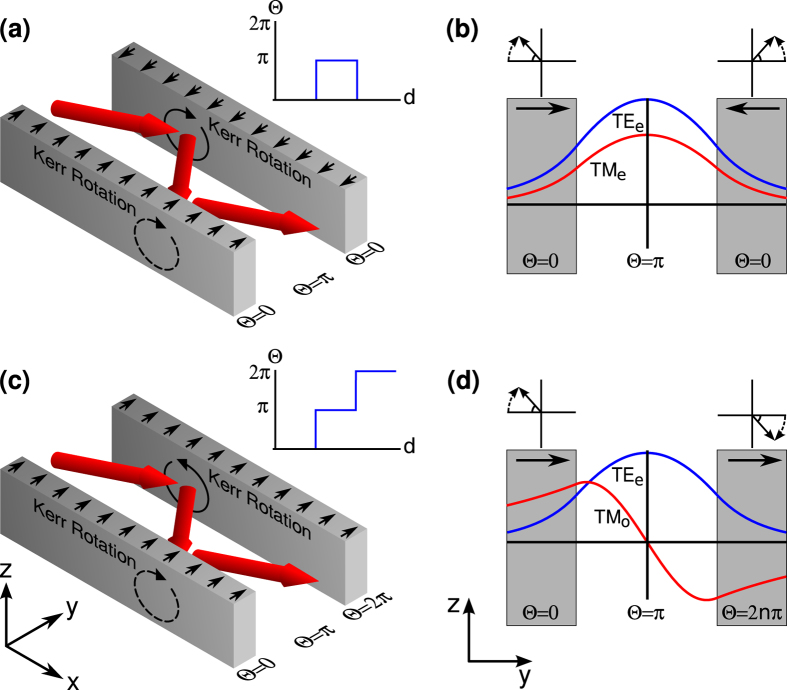
Schematic diagrams of time-reversal-symmetry-broken topological insulator waveguides. (**a**) The anti-parallel configuration. The time-reversal-symmetry-breaking perturbations at each interface of the waveguide are in opposite directions. (inset) The axion coupling profile for this configuration. (**b**) As the time-reversal-symmetry-breaking perturbations at each interface of the waveguide are in opposite directions, the rotation of the magnetic fields at each interface are in the same direction with respect to the propagation direction and, hence, the TE and TM even modes couple. (**c**) The parallel configuration. The time-reversal-symmetry-breaking perturbations at each interface of the waveguide are in the same directions. (inset) The axion coupling profile for this configuration. (**d**) As the time-reversal-symmetry breaking perturbations at each interface of the waveguide are in the same direction, the rotations of the magnetic fields at each interface are in opposite directions with respect to the propagation direction and, hence, the TE even and TM odd modes couple.

There are two possible orientations for the axion coupling. These orientations are a result of the relative direction of the time-symmetry-breaking perturbation on the two sides of the guide layer and originate from the relative sign of the hall conductivities at these interfaces^9^,^10^. The first orientation is anti-parallel orientation [Fig. 1(a)], where the time-symmetry-breaking perturbation is in the opposite direction on each side of the waveguide (and, hence, the change in the axion coupling is the same on each side of the waveguide). In this case the interaction rotates the electromagnetic wave in the same direction with respect to the direction of propagation. Hence, magnetic fields of the same sign (and electric fields of opposite sign) are generated at opposing interfaces. Thus, in a symmetric waveguide, even modes will couple to even modes and odd modes to odd modes [Fig. 1(b)]. The second orientation is the parallel orientation [Fig. 1(c)], where the direction of the time-symmetry-breaking perturbation is in the same on each side of the waveguide (and, hence, the change in the axion coupling is opposite on each side of the waveguide). In this case the interaction rotates the electromagnetic wave in opposite directions with respect to the direction of propagation. Hence, magnetic fields of opposite sign (and electric fields of the same sign) are generated at opposing interfaces. Thus, in a symmetric waveguide, even modes will couple to odd modes vice versa [Fig. 1(d)].

To quantify this effect we use the modified wave equation to develop a coupled mode theory for the system (see Supplementary Material). Owing to the relative sizes of *α* and *ε*, we assume that the axion coupling term only marginally changes the modes of the waveguide and, hence, we can treat it as a small perturbation to the usual magneto-dielectric waveguide modes. Using these modes as a basis, one finds that the mode amplitudes, 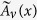, evolve as


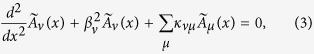


with the coupling constants, *κ*_*νμ*_, give by


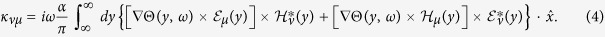


where 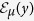 and 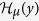 are the usual electric and magnetic field profiles for a magneto-dielectric waveguide and *β*_*ν*_ are the corresponding propagation constants. Here, the indices *μ* and *ν* indicate the TE, TM, odd and even modes. Considering only the lowest four modes in a symmetric waveguide, the interaction only couples pairs of modes, hence, Eq. (3) breaks down into two independent 2 × 2 matrix equations that can be solved analytically.

## Results

### Faraday rotation and ellipticity

As an example, we consider a symmetric waveguide in the anti-parallel configuration with the input electromagnetic wave exciting the TE even mode only. Evaluation of the coupling constants shows that, in this situation, the interaction only couples the input electromagnetic wave to the TM even mode. Solving the resulting system of equations shows that the *E*_*y*_(*x*) and *E*_*z*_(*x*) components of the field evolve as the sum of two waves of different frequencies. The coupled-mode analysis described above leads to two the characteristic parameters for the waveguide, the maximum transferred power, *P*_*c*_, which is the maximum power that the waveguide can transfer from the initial mode (in this case the TE even mode) to the target mode (in this case the TM even mode) and the coupling length, *l*_*c*_, which is the distance that the radiation must travel down the waveguide to achieve this maximum power transfer. In weak coupling limit, 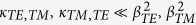, which is always valid for the parameters considered here, one finds


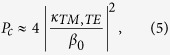


and


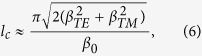


with 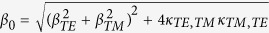. From the field components one can also find the maximum polarization rotation, which occurs at the coupling length, *l*_*c*_, and the maximum ellipticity, which occurs at *l*_*c*_/2. In the weak coupling limit, these read


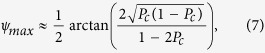



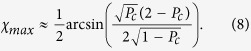


One can quantify the enhancement of the Faraday rotation by comparing Eq. (7) to the result for plane-polarized light at normal incident on a single slab with two interfaces. In this geometry the maximum rotation is achieved in the topological regime^32^ hence the enhancement factor reads *F*_*ψ*_ = *ψ*_*max*_/*α*. For small enhancements this expression simplifies to 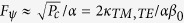.

In order to validate the theoretical model we compared the results to finite-difference-time-domain electromagnetic simulation data^33^. We consider a Bi_2_Se_3_ guide layer, with *ε*_*g*_ = 16^34^, *μ* = 1 and Θ = *π*, and a silicon cladding^12^,^35^, with *ε*_*c*_ = 12, *μ* = 1 and Θ = 0. Bi_2_Se_3_ has a bulk band gap of ≈300 meV^36^,^37^, and an achievable surface gap of ≈100 meV^18^. The strong resonance peak at ≈10 meV^34^ stems from the excitation of surface plasmons^38^, which will be absent in the presence of a surface band gap. Hence, for wavelengths >12 *μm* material absorption will be minimal. Therefore, we focus on the process in the absence of material loss. We insert electromagnetic radiation of wavelength ≈3.3 times the waveguide width. At this frequency there are four guided modes - the lowest order TE and TM, odd and even modes.

**Figure 2:**
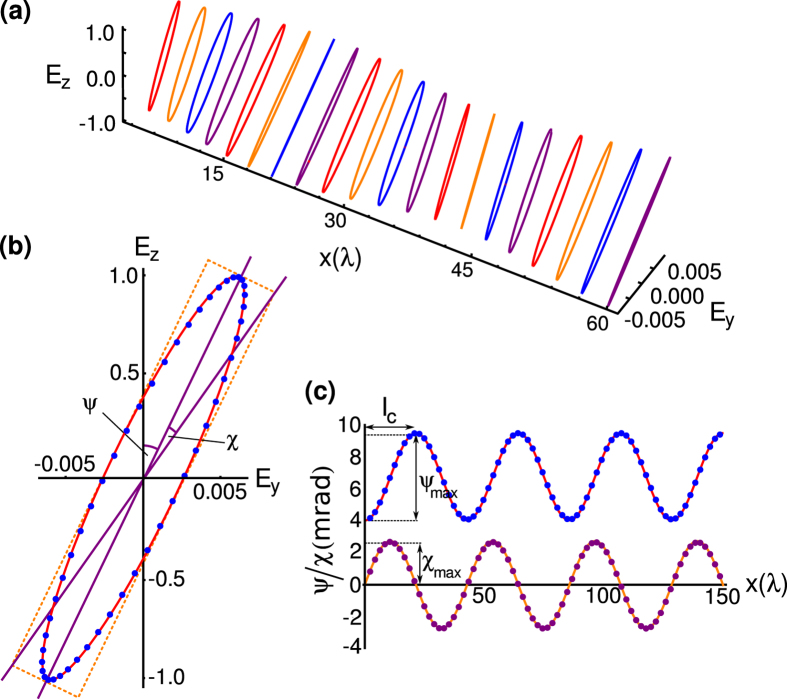
Variation of the polarization angle and ellipticity with distance in a time-reversal-symmetry-broken topological insulator waveguide. (**a**) Simulated data of the polarization and ellipticity of plane polarized light as a function of distance along the waveguide. (**b**) The ellipticity and polarization angle of light at ≈33 wavelengths along the waveguide. The red line shows the theoretically calculated values and the solid blue circles show the simulated data. The angles marked *ψ* and *χ* are the polarization angle and ellipticity, respectively. (**c**) The polarization angle and ellipticity as a function of distance along the waveguide. The red and orange lines are the theoretically calculated values and the solid blue and purple circles are the simulated data. The maximum polarization angle occurs at the coupling length, *l*_*c*_, and the largest ellipticity occurs at half the coupling length, *l*_*c*_/2. The 4 mrad offset in the polarization angle is due to partial excitation of the TM even mode.

Figure 2(a) shows simulated data of the elliptical polarization of the electromagnetic wave at different points along the waveguide. One observes the expected changes in polarization and ellipticity as the wave propagates. Figure 2(b) shows the elliptical polarization of the wave at ≈33 wavelengths along the waveguide for both the theoretical calculation and simulated data. One observes good agreement between the two. Figure 2(c) shows the variation in the polarization angle and ellipticity along the length of the waveguide. The polarization and ellipticity oscillate, with the maximum rotation occurring at *l*_*c*_ and maximum ellipticity occurring at *l*_*c*_/2. The phenomenological shift of 4 mrad that occurs in the simulated data is a result of the source partially exciting the TM even mode hence the initial polarization is 4 mrad away from the z-axis. Good agreement is seen between the numerical calculation and simulated data.

### Permittivity contrast

**Figure 3:**
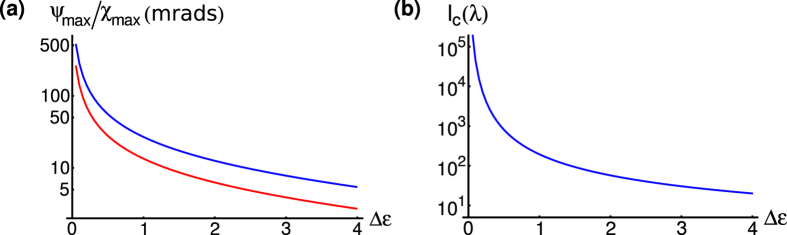
Variation of the polarization angle, ellipticity and coupling length with permittivity contrast in a time-reversal-symmetry-broken topological insulator waveguide. (**a**) Log-linear plot of theoretically calculated values of the variation in the maximum polarization angle, *ψ*_*max*_, (blue line) and ellipticity, *χ*_*max*_, (red line) as a function permittivity contrast. (**b**) Log-linear plot of theoretically calculated values of the variation in the coupling length, *l*_*c*_, as a function of permittivity contrast.

The coupling constants are proportional the waveguide mode profiles, hence, by careful choice of the magneto-dielectric properties of the waveguide guide layer and cladding, the polarization rotation and ellipticity can be maximized. Figure 3(a) shows the maximum polarization rotation and ellipticity for different permittivity contrasts, *ε*_*g*_ − *ε*_*c*_. As the contrast is reduced the maximum polarization rotation and ellipticity increases and, for contrasts of ≈0.5, one sees a rotation of ≈100 mrads (≈6^*o*^), which is two orders of magnitude larger than the rotation on transmission or reflection at a single interface. This increase in rotation is due to the reduced the phase mismatch, *β*_*TE*_ − *β*_*TM*_, between the TE even and TM even modes and, hence, better transfer of energy. However, reducing the permittivity contrast also reduces the coupling constant and, hence, increases the coupling length [see Fig. 3(b)]. Thus, in order to observe the maximal rotation, very long waveguides need to be fabricated (≈1000 wavelengths). For wavelengths below the surface band gap, waveguides on the order of 1–2 cm are required to observe the ≈100 mrads rotation.

## Discussion

Here we have developed a coupled mode theory to describe the propagation of electromagnetic waves in a time-reversal-symmetry-broken topological insulator waveguide. For an axion coupling of Θ = *π* in the anti-parallel configuration and radiation of wavelength *λ* > 12 *μm* (i.e. below the surface band gap) a Faraday rotation of ≈100 mrads (≈6^*o*^) can be achieved for waveguides of length ≈1.2 cm when the permittivity contrast is low. Fabrication of such a device is not infeasible. Current technology for on-chip optical signal processing in silicon is able to produce waveguides in the range of 1–5 cm^39^,^40^. In comparison, fabrication of structures with topological insulators, such as Bi_2_Se_3_, is still in its infancy. The widely used technique of mechanical exfoliation is the current standard and produces micron-sized flakes, which have been successfully used to create simple electronic devices. However, recent advances in epitaxial growth have resulted in Bi_2_Se_3_ thin films with areas of 3 × 3 cm that can be transferred to arbitrary substrates^41^. Whilst the technology to produce cm-long waveguides in Bi_2_Se_3_ is not yet fully developed, the advances in this field show that the fabrication of such a structure is possible and it is envisaged that methods to generate complex heterostructures with topological insulators will be available in the very near future.

**Figure 4:**
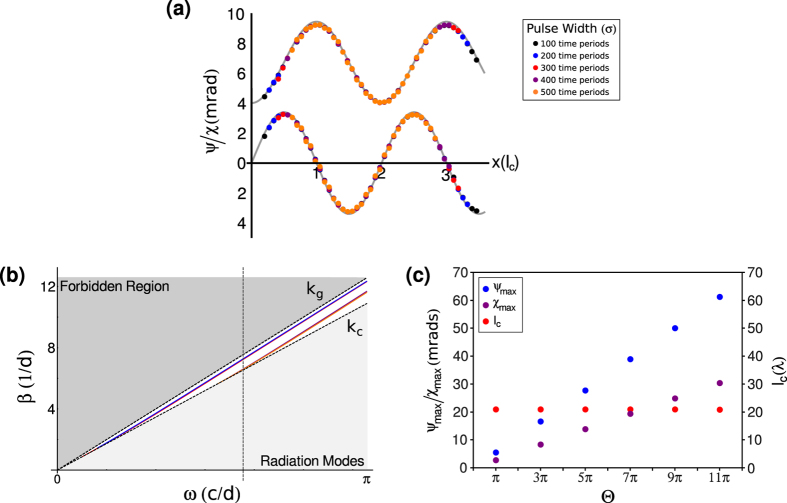
Dispersion and quantization properties of a time-reversal-symmetry-broken topological insulator waveguide. (**a**) Simulation of the polarization rotation and ellipticity for a Gaussian pulse. The solid circles show the simulated data for various pulse widths, where *σ* is the standard deviation of the Gaussian pulse in carrier frequency time periods. The solid grey line are the theoretical calculated values. The waveguide parameters are the same as those used in Fig. 2. (**b**) Theoretically calculated dispersion curve of the waveguide using the same parameters as in Fig. 2. *d* is the waveguide width. The TE even, TM even, TE odd and TM odd modes are represented by the solid blue, red, purple and orange lines respectively. The vertical dashed line indicates the frequency used in the simulations. (**c**) Theoretically calculated values of the polarization rotation (blue circles), ellipticity (purple circles) and coupling length (red circles) for different values of the axion coupling, using the waveguide parameters as in Fig. 2.

When considering the application of a time-reversal-symmetry-broken topological insulator waveguide for control of THz frequency radiation dispersion effects need to be considered. In the previous analysis it was assumed that the electromagnetic radiation was a plane-wave. In more realistic scenarios pulsed light is more commonly used, especially in the field of signal processing. Thus, dispersion becomes an issue. Figure 4(a) shows simulated data of the polarization rotation and ellipticity at the peak of an Gaussian pulse, with temporal envelope function *E*(*t*) = exp[−*t*^2^/*σ*^2^], for various pulse widths, *σ*. The simulations show that the maximum polarization rotation and ellipticity are unchanged by the spectral width and, hence, are almost identical to the theoretically derived result for the continuous wave case.

As the axion coupling, Θ, is 2 orders of magnitude smaller than the permittivity, *ε*, dispersion effects within the waveguide are dominated by magneto-electric properties of the constituent materials. Figure 4(b) shows the allowed propagating waveguide modes as a function of frequency. The mode dispersion curves are almost identical to the equivalent magneto-dielectic waveguide mode dispersion curves vanishing axion coupling, Θ. Hence, the axion coupling only negligibly affects the dispersion properties of the waveguide. Thus a reasonable estimate of the dispersion properties of the waveguide can be found whilst neglecting the effects of the axion coupling.

An important feature of the magneto-electric effect in topological insulators is the universal quantization of the Faraday rotation in units of the fine structure constant, *α*^12^,^13^,^14^,^15^. Clearly, if the polarization rotation is enhanced it will no longer be in units of *α*, however, it is still quantized. The maximum polarization rotation (ellipticity) are quantized in odd multiples of the polarization rotation (ellipticity) for Θ = *π*. Hence, *ψ*_*max*_(Θ = *nπ*) = *nψ*_*max*_(Θ = *π*) and *χ*_*max*_(Θ = *nπ*) = *nχ*_*max*_(Θ = *π*). Thus, by varying the value of the axion coupling, Θ, which can be achieved by varying the time-symmetry breaking perturbation, one would still observe quantized steps as each successive plateau is reached. Figure 4(c) shows the change in maximum polarization rotation and ellipticity with changing axion coupling. One can clearly see the equal steps in the polarization rotation and ellipticity as the axion coupling increases. Note that the axion coupling, as it is directly related to the quantized hall plateaus in the material, can only take odd units of *π* and that a smoothly varying magnetic field will lead to step like transitions in the axion coupling as one access the next hall plateau^9^,^10^.

Whilst this system does not display the universality of the single slab system, the advantage here is that the quantized transitions are much larger. As the polarization rotation is related to electron surface states, the enhanced polarization rotation can be used to probe these states with a greater degree of accuracy. Precision measurements of this type may reveal additional features to these surface states or the transitions between them that will shed light on the quantum processes that occur with topological insulators. Figure 4(c) also shows the variation in the coupling length for varying axion couplings. As the coupling length is more strongly affected by the propagation constants of the modes rather than the axion coupling, one finds that the coupling length remains approximately constant with increasing axion coupling. Thus, one can observe the quantized maximum polarization rotations merely by changing the time-reversal-symmetry breaking perturbation, without the need to change the length of the waveguide.

In terms of potential applications, topological insulators have been proposed as a platform for controlling THz radiation for photodetection^42^, wireless communication and biological spectroscopy^27^. It has also been shown that the Faraday rotation in a topological insulator can be controlled by electronic gating^27^. Thus, it is possible to electrically control the polarization rotation and, hence, provide high-speed optical modulation. Waveguide structures offer a compact method for the guiding of radiation and the continuing development of silicon photonics as a method for optical signal processing has demonstrated the potential of such devices. The integration of topological insulators into existing silicon heterostructure technology would add extra functionality to these photonic components.

Topological insulator waveguides offer an easily fabricable structure that can increase the rotation of plane-polarized electromagnetic radiation, a key signature of the magneto-electric effect, which itself is linked to the electronic surface states of the material, by two orders of magnitude compared to other three-layered structures, thus rendering these features much easier to study. Thus, this effect can be used as a probe of these surface states in fundamental physics experiments. Furthermore, by exploiting the enhancement effects of certain geometries, topological insulator devices, such as the waveguide described here, potentially offer new methods of controlling the polarization of THz electromagnetic radiation.

## Methods

Validation of the theoretical model was carried out by comparison with finite-difference-time-domain electromagnetic simulation data. Using the constitutive equations, Eqs (1) and (2), to eliminate the magnetic induction, **B**(**r**, *ω*), and displacement, **D**(**r**, *ω*), fields from the time dependent Maxwell’s equations and neglecting terms of order *O*(*α*^2^) leads to two coupled equations for the electric, **E**(**r**, *ω*), and magnetic, **H**(**r**, *ω*), fields


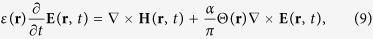



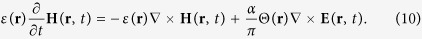


Equations (9) and (10), subject to a unit plane wave source, *E*_*z*_ = −*H*_*y*_ = exp[−*iωt*], were solved on a staggered Yee lattice using standard electromagnetic finite-difference-time-domain methods^33^.

## Additional Information

**How to cite this article**: Crosse, J. A. Theory of topological insulator waveguides: polarization control and the enhancement of the magneto-electric effect. *Sci. Rep.* **7**, 43115; doi: 10.1038/srep43115 (2017).

**Publisher's note:** Springer Nature remains neutral with regard to jurisdictional claims in published maps and institutional affiliations.

## Supplementary Material

Supplementary Material
