# Supplementary material for: Theory of topological insulator waveguides: polarization control and the enhancement of the magneto-electric effect
Source: Sci Rep. 2017 Feb 21;7:43115. doi: 10.1038/srep43115 (PMC5318882; doi:10.1038/srep43115)
Supplement: Supplementary Material [file srep43115-s1.pdf]

# SUPPLEMENTARY MATERIAL for Theory of topological insulator waveguides: polarization control and the enhancement of the magneto-electric effect

**J. A. Crosse**[1,*]

[1]Department of Electrical and Computer Engineering, National University of Singapore, 4 Engineering Drive 3, Singapore 117583.
[*]alexcrosse@gmail.com

Consider a slab waveguide consisting of three layers, layered in the y-direction, with permittivity profile, $\varepsilon(y,\omega)$, permeability profile, $\mu(y,\omega)$, and axion coupling profile, $\Theta(y,\omega)$. We arrange our coordinate system such that the guided modes propagate in the x-direction. From the source free Maxwell Equations and constitutive relations for a time-reversal-symmetry-broken topological insulator, one can show that, in the presence of a non-vanishing axion coupling, the electric, $\mathbf{E}(\mathbf{r},\omega)$, and magnetic, $\mathbf{H}(\mathbf{r},\omega)$, fields obey the wave equations

$$\nabla^2\mathbf{E}(\mathbf{r},\omega)+\omega^2\varepsilon(y,\omega)\mathbf{E}(\mathbf{r},\omega)+i\omega\frac{\alpha}{\pi}\left[\boldsymbol{\nabla}\Theta(y,\omega)\times\mathbf{E}(\mathbf{r},\omega)\right]=\mathbf{0}, \tag{1}$$

$$\nabla^2\mathbf{H}(\mathbf{r},\omega)+\omega^2\varepsilon(y,\omega)\mathbf{H}(\mathbf{r},\omega)+i\omega\frac{\alpha}{\pi}\left[\boldsymbol{\nabla}\Theta(y,\omega)\times\mathbf{H}(\mathbf{r},\omega)\right]=\mathbf{0}, \tag{2}$$

respectively. Here, for convenience, we work in natural units where $c=\varepsilon_0=\mu_0=1$ and we have assumed that magnetic effects are negligible and, hence, $\mu(y,\omega)=1$. Furthermore, as the fine structure constant, $\alpha$, is of the order $10^{-2}$ compared to the permittivity, $\varepsilon(y,\omega)$, terms of order $\alpha^2$ are also negligible.

Owing to the relative sizes of $\alpha$ and $\varepsilon$, we assume that the axion coupling only marginally changes the modes of the waveguide and hence we can treat the last terms in Eqs. (1) and (2) as a small perturbation to the usual waveguide modes of a traditional magneto-dielectric waveguide. Hence we can expand the electric and magnetic field as

$$\mathbf{E}(\mathbf{r},\omega)=\sum_\mu A_\mu(x)\mathscr{E}_\mu(y)e^{i\beta_\mu x}, \tag{3}$$

$$\mathbf{H}(\mathbf{r},\omega)=\sum_\mu A_\mu(x)\mathscr{H}_\mu(y)e^{i\beta_\mu x}, \tag{4}$$

where $\mathscr{E}_\mu(y)$ and $\mathscr{H}_\mu(y)$ are the usual electric and magnetic field profiles of mode $\mu$ for a magneto-dielectric waveguide and $\beta_\mu$ and $A_\mu(x)$ are the corresponding propagation constant and amplitude. Substituting the expansions in Eqs. (3) and (4) into Eqs. (1) and (2) and noting that, by definition, the dielectric waveguide modes obey

$$\frac{\partial^2}{\partial y^2}\mathscr{E}_\mu(y)+\left[\omega^2\varepsilon(y,\omega)-\beta_\mu^2\right]\mathscr{E}_\mu(y)=\mathbf{0}, \tag{5}$$

$$\frac{\partial^2}{\partial y^2}\mathscr{H}_\mu(y)+\left[\omega^2\varepsilon(y,\omega)-\beta_\mu^2\right]\mathscr{H}_\mu(y)=\mathbf{0}, \tag{6}$$

one finds that the wave equations become

$$\sum_\mu\left[\frac{\partial^2}{\partial x^2}\tilde{A}_\mu(x)+\beta_\mu^2\tilde{A}_\mu(x)\right]\mathscr{E}_\mu(y)+i\omega\frac{\alpha}{\pi}\sum_\mu\left[\boldsymbol{\nabla}\Theta(y,\omega)\times\mathscr{E}_\mu(y)\right]\tilde{A}_\mu(x)=\mathbf{0}, \tag{7}$$

$$\sum_\mu\left[\frac{\partial^2}{\partial x^2}\tilde{A}_\mu(x)+\beta_\mu^2\tilde{A}_\mu(x)\right]\mathscr{H}_\mu(y)+i\omega\frac{\alpha}{\pi}\sum_\mu\left[\boldsymbol{\nabla}\Theta(y,\omega)\times\mathscr{H}_\mu(y)\right]\tilde{A}_\mu(x)=\mathbf{0}, \tag{8}$$

where $\tilde{A}_\mu(x)=\exp\left[i\beta_\mu x\right]\tilde{A}_\mu(x)$. Using the orthogonality relation

$$\int_{-\infty}^{\infty}dy\left[\mathscr{E}_\mu(y)\times\mathscr{H}^*_\nu(y)+\mathscr{E}^*_\nu(y)\times\mathscr{H}_\mu(y)\right]\cdot\hat{x}=\delta_{\mu\nu}, \tag{9}$$

where $\delta_{\mu\nu}$ is the Kronecker delta, one can show that the coupled mode equations for a specific mode, $\tilde{A}_\nu(x)$, read

$$\frac{d^2}{dx^2}\tilde{A}_\nu(x)+\beta_\nu^2\tilde{A}_\nu(x)+\sum_\mu \kappa_{\nu\mu}\tilde{A}_\mu(x)=0, \tag{10}$$

with the coupling constant $\kappa_{\nu\mu}$ is defined as

$$\kappa_{\nu\mu}=i\omega\frac{\alpha}{\pi}\int_\infty^\infty dy\left\{\left[\mathbf{\nabla}\Theta(y,\omega)\times\mathscr{E}_\mu(y)\right]\times\mathscr{H}_\nu^*(y)+\left[\mathbf{\nabla}\Theta(y,\omega)\times\mathscr{H}_\mu(y)\right]\times\mathscr{E}_\nu^*(y)\right\}\cdot\hat{x}. \tag{11}$$

For a slab waveguide with a guide layer of width, $d$, centered at $y=0$ and constant axion couplings $\Theta_+$, $\Theta_-$ and $\Theta_g$, in the upper, lower and guide layers respectively, one can write the overall axion coupling as

$$\Theta(y,\omega)=\Theta_- H\left(-y-\frac{d}{2}\right)+\Theta_g H\left(y+\frac{d}{2}\right)H\left(-y+\frac{d}{2}\right)+\Theta_+ H\left(y-\frac{d}{2}\right), \tag{12}$$

where $H(y)$ is the Heaviside step function. The derivative of the axion coupling profile reads

$$\mathbf{\nabla}\Theta(y,\omega)=(\Theta_g-\Theta_-)\delta\left(y+\frac{d}{2}\right)-(\Theta_g-\Theta_+)\delta\left(y-\frac{d}{2}\right). \tag{13}$$

Thus, one sees that the coupling term vanishes everywhere except at the interface of the guide layer and the cladding. Substituting, Eq. (13) into the definition of the coupling constant in Eq. (11) and evaluating the cross products leads to

$$\kappa_{\nu\mu}=i\beta_\nu\frac{\alpha}{\pi}(\Theta_g-\Theta_+)\left\{\frac{1}{\varepsilon_g}\mathscr{H}_{\nu,z}^*\left(\frac{d}{2}\right)\mathscr{H}_{\mu,x}\left(\frac{d}{2}\right)-\mathscr{E}_{\nu,z}^*\left(\frac{d}{2}\right)\mathscr{E}_{\mu,x}\left(\frac{d}{2}\right)\right\}$$
$$-i\beta_\nu\frac{\alpha}{\pi}(\Theta_g-\Theta_-)\left\{\frac{1}{\varepsilon_g}\mathscr{H}_{\nu,z}^*\left(-\frac{d}{2}\right)\mathscr{H}_{\mu,x}\left(-\frac{d}{2}\right)-\mathscr{E}_{\nu,z}^*\left(-\frac{d}{2}\right)\mathscr{E}_{\mu,x}\left(-\frac{d}{2}\right)\right\}, \tag{14}$$

where $\varepsilon_g$ is the permittivity of the guide layer. This shows that the effect of the axion coupling only occurs at the waveguide boundary and its action is to rotate the transverse electric and magnetic field vectors, with the strength of the interaction proportional to the change in the axion coupling and the overlap of the waveguide modes at the interface.

There are two possible orientations for the axion coupling, the parallel orientation, where the change in the axion coupling is in opposite directions at each interface, $\mathrm{sgn}(\Theta_g-\Theta_+)=-\mathrm{sgn}(\Theta_g-\Theta_-)$, and the anti-parallel orientation, where the change in the axion coupling is in the same direction at each interface, $\mathrm{sgn}(\Theta_g-\Theta_+)=\mathrm{sgn}(\Theta_g-\Theta_-)$. The example in the main text has $\Theta_g=\pi$ and $\Theta_-=\Theta_+=0$. These values satisfy $\mathrm{sgn}(\Theta_g-\Theta_+)=\mathrm{sgn}(\Theta_g-\Theta_-)$ and hence this is the anti-parallel configuration. Conversely, $\Theta_g=\pi$, $\Theta_-=0$ and $\Theta_+=2\pi$ satisfies $\mathrm{sgn}(\Theta_g-\Theta_+)=-\mathrm{sgn}(\Theta_g-\Theta_-)$ and this is the parallel configuration. In principle, there is one further situation where $\Theta_g=\Theta_-=\Theta_+=0$. In this case both $\mathrm{sgn}(\Theta_g-\Theta_+)=\mathrm{sgn}(\Theta_g-\Theta_-)$ and $\mathrm{sgn}(\Theta_g-\Theta_+)=-\mathrm{sgn}(\Theta_g-\Theta_-)$ are satisfied. However, this is the case where neither the guide layer nor the cladding are topological insulators (i.e. we have a normal magneto-dielectric waveguide) and, hence, the system is topologically trivial and the coupling constant vanishes.

Evaluation of the coupling constants shows that for a symmetric waveguide in the parallel configuration the TE even modes couple to the TM odd modes and the TE odd modes couple to the TM even modes. In a symmetric waveguide in the anti-parallel configuration the TE even modes couple to the TM even modes and the TE odd modes couple to the TM odd modes. In both cases the TE even and odd and TM even and odd modes do not couple and there is no self-coupling. Thus, if one considers only the lowest four modes in each configuration, the four coupled mode equations can be split into two independent sets of two coupled mode equations. In the following we focus on the anti-parallel configuration. All other configurations follow identically with the appropriate replacement of mode profiles and coupling constants. In this situation, only the even modes are relevant and the coupled mode equations in Eq. (10) reduce to

$$\frac{d^2}{dx^2}\begin{pmatrix}A_{TE}\\A_{TM}\end{pmatrix}+\begin{pmatrix}\beta_{TE}^2 & \kappa_{TE,TM}\\ \kappa_{TM,TE} & \beta_{TM}^2\end{pmatrix}\begin{pmatrix}A_{TE}\\A_{TM}\end{pmatrix}=0. \tag{15}$$

Solving Eq. (15) in the diagonal basis and reverting to the mode basis leads to

$$\begin{pmatrix}A_{TE}(x)\\A_{TM}(x)\end{pmatrix}=\frac{1}{\beta_0}\begin{pmatrix}(\lambda_+^2-\beta_{TM}^2)e^{i\lambda_+x}-(\lambda_-^2-\beta_{TM}^2)e^{i\lambda_-x} & \kappa_{TE,TM}\left(e^{i\lambda_+x}-e^{i\lambda_-x}\right)\\ \kappa_{TM,TE}\left(e^{i\lambda_+x}-e^{i\lambda_-x}\right) & (\lambda_+^2-\beta_{TE}^2)e^{i\lambda_+x}-(\lambda_-^2-\beta_{TE}^2)e^{i\lambda_-x}\end{pmatrix}\begin{pmatrix}A_{TE}(0)\\A_{TM}(0)\end{pmatrix}=0, \tag{16}$$

with the eigenvalues of the system reading

$$\lambda_{\pm}^2 = \frac{\beta_{TE}^2 + \beta_{TM}^2 \pm \beta_0}{2}, \tag{17}$$

with

$$\beta_0 = \sqrt{(\beta_{TE}^2 + \beta_{TM}^2)^2 + 4\kappa_{TE,TM}\kappa_{TM,TE}}. \tag{18}$$

If one assumes that at $x = 0$ the source is polarized in the TE mode ($A_{TE}(0) = 1$ and $A_{TM}(0) = 0$) one can see from Eq. (16) that the power in the TM mode at a distance $x$ is given by

$$A_{TM}(x) = \frac{\kappa_{TE,TM}}{\beta_0}\left(e^{i\lambda_+ x} - e^{i\lambda_- x}\right), \tag{19}$$

which consists of combination of two waves with wavelengths $\lambda_-$ and $\lambda_+$. The maximum power that can be transferred to the TM mode is given by the maximum value of the expression in Eq. (19) and can be found to be

$$P_c = 4\left|\frac{\kappa_{TE,TM}}{\beta_0}\right|^2. \tag{20}$$

The distance along the waveguide that the electromagnetic wave must travel to achieve the maximum power transfer is known as the coupling length and is given by

$$l_c = \frac{\pi}{\lambda_+ - \lambda_-} \approx \frac{\pi\sqrt{2(\beta_{TE}^2 + \beta_{TM}^2)}}{\beta_0}, \tag{21}$$

where the approximate solution is valid in weak coupling limit, $\kappa_{TE,TM}, \kappa_{TM,TE} \ll \beta_{TE}^2, \beta_{TM}^2$, which is always valid for the parameters considered here.

Given the electric field components, the polarization angle, $\psi$, and ellipticity, $\chi$, are given by

$$\psi = \frac{1}{2}\arctan\left(\frac{2|E_z|^2}{|E_z|^2 - |E_y|^2}\mathrm{Re}\left[\frac{E_y}{E_z}\right]\right), \tag{22}$$

$$\chi = \frac{1}{2}\arcsin\left(\frac{2|E_z|^2}{|E_z|^2 + |E_y|^2}\mathrm{Im}\left[\frac{E_y}{E_z}\right]\right). \tag{23}$$

The maximum polarization rotation occurs at the coupling length, $l_c$, and the maximum ellipticity occurs at half the coupling length, $l_c/2$, and can be found from Eqs. (21), (20), (22) and (23). In the weak coupling limit these quantities read

$$\psi \approx \frac{1}{2}\arctan\left(\frac{2\sqrt{P_c(1-P_c)}}{1-2P_c}\right), \tag{24}$$

$$\chi \approx \frac{1}{2}\arcsin\left(\frac{\sqrt{P_c}(2-P_c)}{2\sqrt{1-P_c}}\right), \tag{25}$$

One can see that both the polarization rotation and ellipticity are a function of the maximum transferred power, which is a function of the waveguide structure. Thus, a specific waveguide will lead to a specific polarization rotation and ellipticity.
